# A data-driven spatial approach to characterize the flood hazard

**DOI:** 10.3389/fdata.2022.1022900

**Published:** 2022-12-12

**Authors:** Rubayet Bin Mostafiz, Md Adilur Rahim, Carol J. Friedland, Robert V. Rohli, Nazla Bushra, Fatemeh Orooji

**Affiliations:** ^1^Department of Oceanography and Coastal Sciences, College of the Coast and Environment, Louisiana State University, Baton Rouge, LA, United States; ^2^Coastal Studies Institute, Louisiana State University, Baton Rouge, LA, United States; ^3^LaHouse Resource Center, Department of Biological and Agricultural Engineering, Louisiana State University Agricultural Center, Baton Rouge, LA, United States; ^4^Engineering Science Program, Louisiana State University, Baton Rouge, LA, United States; ^5^School of Engineering and Applied Sciences, Western Kentucky University, Bowling Green, KY, United States

**Keywords:** annual exceedance probability (AEP), Gumbel extreme value distribution, spatial interpolation techniques, Special Flood Hazard Area (SFHA), Federal Emergency Management Agency (FEMA), flood risk, shaded X Zone, unshaded X Zone

## Abstract

Model output of localized flood grids are useful in characterizing flood hazards for properties located in the Special Flood Hazard Area (SFHA—areas expected to experience a 1% or greater annual chance of flooding). However, due to the unavailability of higher return-period [i.e., recurrence interval, or the reciprocal of the annual exceedance probability (AEP)] flood grids, the flood risk of properties located outside the SFHA cannot be quantified. Here, we present a method to estimate flood hazards that are located both inside and outside the SFHA using existing AEP surfaces. Flood hazards are characterized by the Gumbel extreme value distribution to project extreme flood event elevations for which an entire area is assumed to be submerged. Spatial interpolation techniques impute flood elevation values and are used to estimate flood hazards for areas outside the SFHA. The proposed method has the potential to improve the assessment of flood risk for properties located both inside and outside the SFHA and therefore to improve the decision-making process regarding flood insurance purchases, mitigation strategies, and long-term planning for enhanced resilience to one of the world's most ubiquitous natural hazards.

## Introduction

The perilous and expensive nature of flood hazards calls for concurrent improvements in the ability of scientists to measure their risk (Kron, 2005). Moreover, rapid increases in the population living in marginal areas relative to the flood hazards (Moulds et al., 2021), amid the consequences of land use changes such as in Bangladesh (Dewan et al., 2007), Belgium (Akter et al., 2018), India (Guhathakurta et al., 2011), China (Shen et al., 2021), the United States (Qiang et al., 2017), and elsewhere, a changing climate (Zhou et al., 2012; Kreibich et al., 2015), sea level rise (Nicholls et al., 1999; Bushra et al., 2021), and local factors such as subsidence (Mostafiz et al., 2021a) and extreme weather events (Guhathakurta et al., 2011), underline the urgent need for accelerated improvements in flood risk assessment (Merz et al., 2014; Mostafiz, 2022). Yet proportionately little advancement has been made. Flood risk maps are often outdated and ignore expression of uncertainty in the depth-duration vs. return period [i.e., recurrence interval, or the reciprocal of the annual exceedance probability (AEP)] relationships (Hassini and Guo, 2017; Tuyls et al., 2018). Consequences of this gap in scientific analysis ripple into many facets of flood awareness, communication, modeling, planning, preparation, and recovery (Huang and Xiao, 2015). Thus, improved quantification of flood hazards, and therefore flood risk, is crucial not only for its own sake, but also for the benefit of other, related efforts to reduce flood-induced losses to life and property (Merz et al., 2014; Mostafiz et al., 2021b, 2022a; Al Assi et al., 2022a; Gnan et al., 2022a; Rahim et al., 2022a).

One component of flood hazard quantification that is of particular importance in planning for development is the accurate estimations of return-period-based flood depths (Yang et al., 2020). This is especially important for infrastructure that is expected to be protected during its service over a long period of usefulness (Requena et al., 2013), such as residential and commercial construction, roads, bridges, tunnels, and historical/cultural sites. Not only do lives and livelihoods depend on the protection of such flood-safe infrastructure (Wiering, 2019), but renovating and rebuilding these resources after a flood is expensive, disruptive, unpleasant, and incongruent with the ongoing quest for healthier and more resilient individuals and communities (Sayers et al., 2018), if it is possible at all.

Not surprisingly given the paucity of updated scientific work on flood, few if any historical records of such estimates may exist to guide construction, protection, or restoration efforts. Thus, reliance on hydrologic and hydraulic modeling of flood events as a function of AEP is necessary (Mostafiz et al., 2021c). However, relatively flood-safe areas often have “null” (i.e., zero or negative) depth values at modeled return periods, even while vulnerability remains substantial during the life span of the infrastructure (Mostafiz et al., 2021c). This leaves even fewer known depth values for planning purposes and may compound flood estimation errors at successively longer return periods, which further weakens efforts to mitigate the impacts of the most destructive floods (Kundzewicz et al., 2013). Therefore, stochastic statistical methods are vital tools to enhance the hydrologic-modeled data for estimating flood (McCuen, 2016), to provide construction specialists, architects, developers, and urban and regional planners with adequate information to build more resilient facilities and communities (Olsen et al., 2015).

Previous research has focused on estimating flood hazard and risk for properties located inside the Special Flood Hazard Area (SFHA—areas exposed to 1% or greater annual chance of flooding), where flood insurance is mandatory (e.g., Posey and Rogers, 2010; Habete and Ferreira, 2017; Johnston and Moeltner, 2019; Mobley et al., 2021). The areas outside the SFHA are divided into the “shaded X Zone” (i.e., between 1 and 0.2% annual chance of flooding inundation areas) and the “unshaded X Zone” (i.e., outside of the 0.2% annual chance of flooding inundation area) (Crowell et al., 2010). Generally, no estimates of flood risk exist for properties located in the shaded or unshaded X Zones (Czajkowski et al., 2013). Additionally, flood insurance is not mandatory in these areas (Kousky, 2018), despite the fact that the flood risk is non-zero, may be substantial (especially where valuable and/or expensive infrastructure exists), and may be poorly understood by scientists (Czajkowski et al., 2013). The properties inside the shaded X Zone are considered to have “moderate” flood risk whereas properties inside the unshaded X Zone are labeled as being subjected to “minimal” flood risk (FEMA, 2005), even though the precise risk throughout the zone is currently unknown. The need for greater quantitative techniques is obvious, so that citizen constituents and government leaders are more aware of the risks that they and their communities face (Mostafiz et al., 2022b).

The overarching goal of this research is to characterize flood hazards at locations both inside and outside the SFHA. More specifically, the research addresses the question, “If no modeled flood data exist for some or all return periods, what are the flood characteristics?” To that end, this research introduces a method for describing flood hazards whereby the flood is characterized using the Gumbel extreme value distribution (Waylen and Woo, 1982; Nadarajah and Kotz, 2004; Al Assi et al., 2022b), and flood elevations are projected at higher return periods (Mostafiz et al., 2021c). The gaps in flood surfaces due to limited data are filled by spatial interpolation techniques. These filled elevation values are then used to estimate floods for the locations inside the shaded or unshaded X Zones.

The contribution of this research is the development of a novel method to estimate flood hazard characteristics based on existing hydrologic-modeled flood surfaces. Ultimately, this technique will help government agencies and community officials to formulate policies and homeowners to make more informed decisions regarding insurance purchase (Rahim et al., 2021, 2022b), mitigation strategy (Zhou et al., 2012; Zarekarizi et al., 2020), and long-term planning (Gnan et al., 2022b,c).

## Method

**Figure 1:**
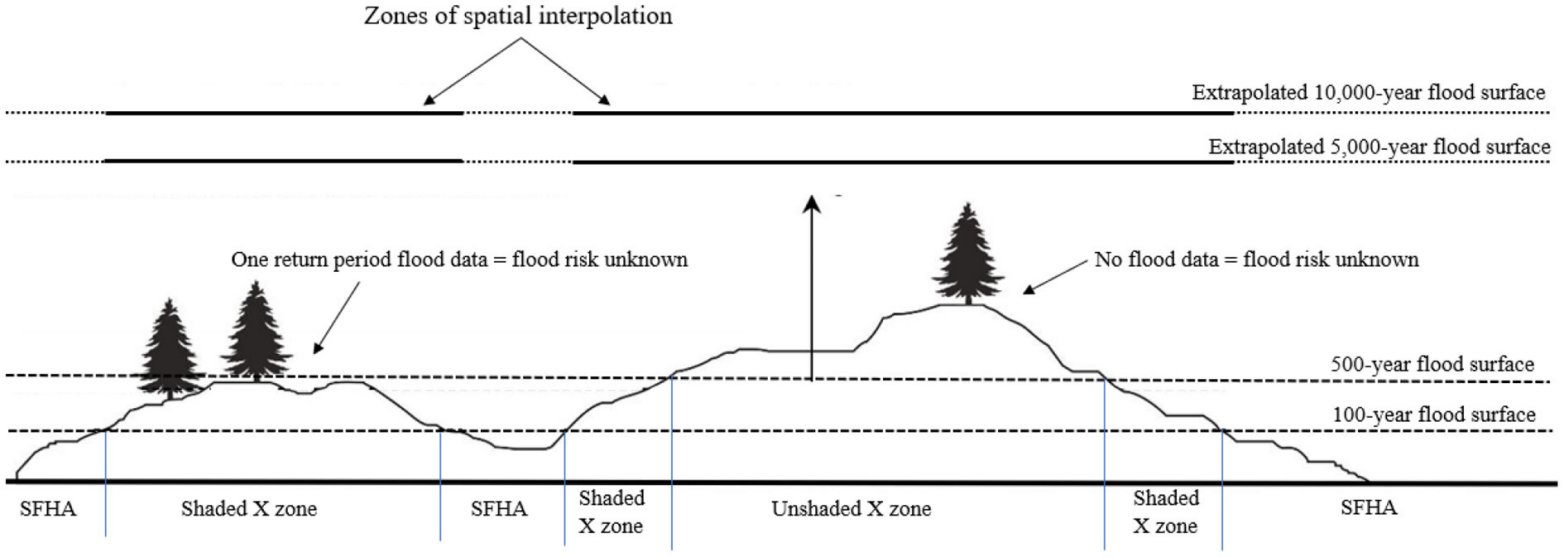
Schematic representation of the concept behind the flood depth surface estimating method.

**Figure 2:**
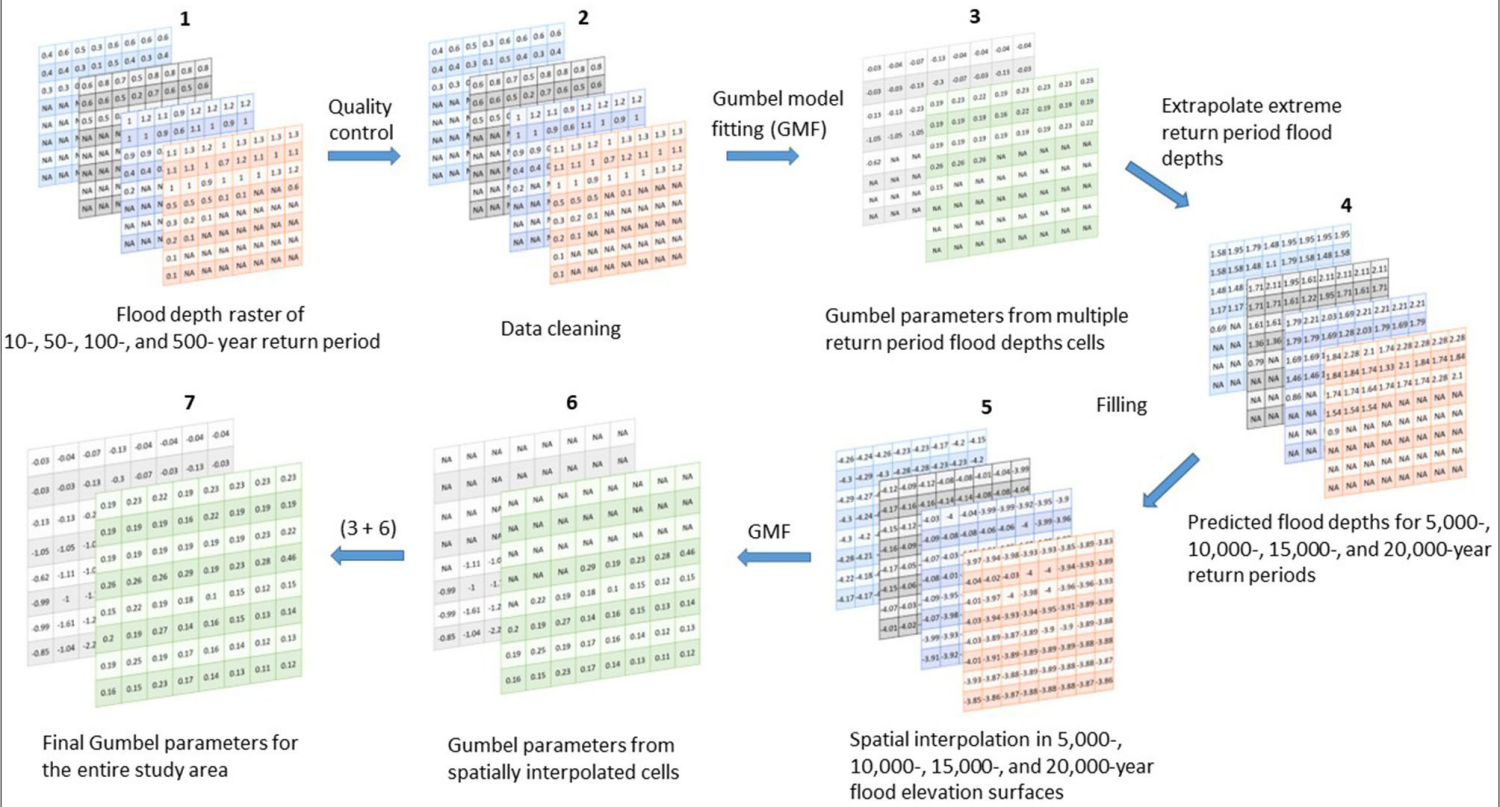
Schematic summary of the flood hazard characterization method.

The method consists of extrapolating flood depths using the Gumbel extreme value distribution at the locations where a Gumbel fit is possible because flood depths for at least two return periods are known. Extreme return periods are selected where most of the study area is assumed to be submerged (Figure 1). Then, spatial interpolation techniques (Lam, 1983; Dewan, 2013), including moving average (e.g., Haining, 1978; Chang et al., 1984), inverse distance weighting (IDW; e.g., Fassnacht et al., 2003; Lu and Wong, 2008), natural neighbor (e.g., Watson, 1999; da Silva et al., 2019), and kriging (e.g., Delhomme, 1978; Oliver and Webster, 1990), are used to estimate the flood elevation for the extreme return periods at grid cells for which no data-derived distribution can be fit confidently. It is necessary to use flood elevation rather than flood depth for spatial interpolation because flood depth cannot be smoothed across space, while flood elevation is generally insensitive to differences in surface elevation. The imputed extreme-return-period flood elevations are then fit with the Gumbel distribution and used to estimate flood depth for locations that are unflooded at shorter return periods to verify that negative values, confirming that the surface is not flooded at that return period) are returned. Through this method, the flood depth vs. annual non-exceedance probability relationships are established for all locations in the study area, which can then be used to develop flood hazard estimates that are more reasonable to expect within the useful life of the building or settlement. The overall schematic summary of the flood hazard characterization method is shown in Figure 2.

### Study area and data

**Figure 3:**
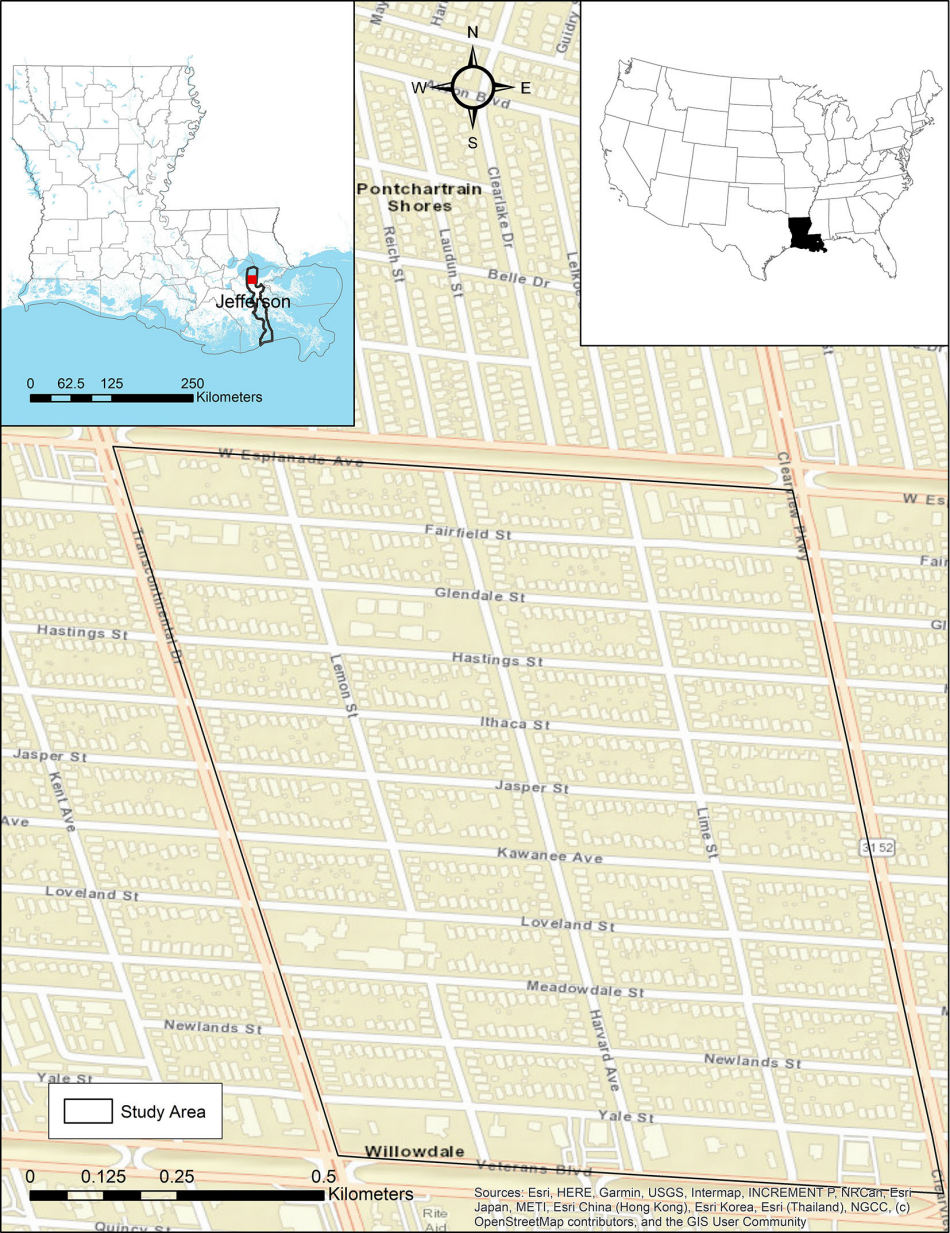
Study area in Metairie, Louisiana.

A frequently-flooded residential neighborhood in Metairie, Louisiana (Jefferson Parish), bounded by the area shown in Figure 3, is used for this case study. This site is chosen primarily because of the availability of model-output flood depth grids for four return periods-−10, 50, 100, and 500 years—developed at a scale of 3.048 × 3.048 m, by FEMA through its Risk Mapping, Assessment and Planning (Risk MAP) program (FEMA, 2021). Although recent research has noted issues with FEMA methodologies and has enhanced flood characterization (Wing et al., 2017, 2018; Bates et al., 2021), these data are considered here due to the wide availability in the United States.

The grid cells located within SFHA have at least two flood depth values (i.e., 100– and 500-year return periods) for which the Gumbel distribution can be fit initially (described in Section 2.3). For the grid cells located in shaded-X zone (i.e., only 500-year flood depth is available) or unshaded-X zone (i.e., no flood information available), spatial interpolation is conducted to characterize flood in these grids (described in Section 2.4).

The study area consists of 44 census blocks with a total area of ~1.126 km^2^. The mean elevation in this below-sea-level, levee-protected area is −5.5 feet with a standard deviation of 0.71 and a range of −9.0 to −2.9 feet. Descriptive statistics of the Risk MAP-output flood depths by return period are shown in Table 1. The spurious maximum value for the 100-year return period, which is equal to that of the 500-year return period (Table 1), suggests that data cleanup is necessary.

**Table 1:** Descriptive statistics of preliminary (uncleaned) flood depths (feet) by return period for the Metairie, Louisiana, study area.

**Return period (years)**	**Mean (ft.)**	**Standard deviation (ft.)**	**Minimum (ft.)**	**Maximum (ft.)**	**Number of flooded cells**
10	0.67	0.45	0.00	3.40	51,937
50	0.75	0.50	0.00	3.70	68,937
100	0.90	0.58	0.00	4.10	91,163
500	0.93	0.58	0.00	4.10	100,705

### Data cleaning

Initial quality checks of the source data are performed to identify cells with unrealistic flood depths. The three types of spurious source data are: (1) any cell with a reported flood depth less than or equal to zero for any return period; (2) any cell in which a flood depth for a shorter return period equals or exceeds that for any longer return period; and (3) any cell in which a shorter-duration return period has a reported flood depth but a longer return period has a null (i.e., flood-free) value. Flood depth values for all return periods at any cell that violate any of the three rules above are characterized as “missing.” Flood depth values for cells in which the depth is known (i.e., non-null) for only the 500-year return period are removed here temporarily, but the regression parameters derived are used later to project flood depth as a function of return period for such cells.

### Gumbel fitting for cells flooded by 100-year return period event

The Gumbel distribution is a widely accepted method for flood frequency analysis (e.g., Kumar and Bhardwaj, 2015; Singh et al., 2018). The right-skewed nature of flood return periods makes the Gumbel distribution ideal for estimating the depth vs. annual non-exceedance probability relationship. The Gumbel distribution has been shown to provide the best fit among extreme value distributions, particularly for smaller sample sizes (World Meteorological Organization, 1989; Onen and Bagatur, 2017).

The Gumbel extreme value probability density function (PDF) as a function of flood depth (*D*) is expressed as:


(1) $$\begin{array}{l} f\left(D\right)=\;\left(\frac{1}{\alpha }\right)\exp\left\{-\left(\frac{D-u}{\alpha }\right)-exp\left[-\left(\frac{D-u}{\alpha }\right)\right]\right\} \end{array}$$

where α and *u* are the calculated, site-specific scale and location parameters, respectively.

The cumulative distribution function (CDF) is equal to the non-exceedance probability, *P*, or


(2) $$\begin{array}{l} P=F\left(D\right)=\;\exp\left\{-exp\left[-\left(\frac{D-u}{\alpha }\right)\right]\right\} \end{array}$$

Solving for *D* yields the Gumbel inverse CDF, where *D* is obtained as a function of *P* and the Gumbel parameters as:


(3) $$\begin{array}{l} {D=F}^{-1}\left[F\left(D\right)\right]=u-\alpha \;\left\{ln\left[-ln\left(P\right)\right]\right\} \end{array}$$

For each cell having non-null *D* for at least two return periods, all non-null return periods are used to fit the Gumbel distribution. The site-specific *u* represents the *D* at a theoretical, asymptotic ~1.58-year return period. Thus, *u* would be positive for cells located in coastal areas or water bodies and negative for cells located in non-water bodies, including residential areas (Mostafiz et al., 2021c), because a developed area would rarely flood at a 1.58-year return period.

The cells that flood at all four (i.e., 10-, 50-, 100-, and 500-year) return periods are examined first. Such cells that represent a water body are distinguished from those that represent a (flood-prone) terrestrial surface. Each cell that is actually terrestrial and has a negative *u* is considered to have a plausible Gumbel fit, while each terrestrial cell with a positive *u* is considered to have a spurious fit. To correct the fit for the cells having a spurious *u* value, the Gumbel distribution is re-fit while including a “dummy” 2-year return period having a *D* of −0.05 feet in addition to the known return period depths. A return period of less than two years is cumbersome because calculation of the natural logarithm function for such short return periods yields an unstable result that approaches negative infinity for near-zero return periods. For each cell in which the resulting re-calculated *u* value based on (now) five return periods then has the appropriate sign, the re-fit Gumbel parameters are accepted. However, for each terrestrial cell in which the re-fit Gumbel distribution again produces a *u* value with a spurious sign, the iteration of re-fitting the Gumbel distribution (this time using a *D* of −0.10 feet) is continued, with the process repeated using incremental dummy decreases in *D* of −0.15 feet, −0.20 feet, etc., with the process ending at the first iteration that generates a negative value for *u*.

The cells flooded at only three (i.e., 50-, 100-, and 500-year) return periods and having null *D* (i.e., flood-free) at the shortest (i.e., 10-year) return period are treated next. For such cells, the Gumbel distribution is fit using only the three valid return periods, and *D* must be estimated for the 10-year return period using the Gumbel distribution with the α and *u* parameters derived for that cell. If such an estimate yields a negative value for the 10-year return period, the estimation is considered valid. However, if the calculation results in a positive value, correction is necessary because the cell is known to be flood-free at that return period. In such cases, a dummy 10-year return period *D* of −0.05 feet is assigned, and the Gumbel distribution is fit once again, this time using this dummy *D*, along with the output for the three *D* values for the same cell. For cells in which this new Gumbel fit using the dummy value produces a “correct” condition (i.e., flood-free) regarding *D*, the revised α *and u* Gumbel parameters are accepted for that cell. However, for cells in which the “correct” flood condition is still not predicted accurately, the dummy 10-year return period *D* is replaced by −0.10 feet, and the Gumbel distribution is then run a third time for that cell. For cells in which this new dummy *D* now generates a “correct” condition, the re-revised α and *u* parameters are “accepted” for that cell, but for those “null” cells still having a positive calculated 10-year-return-period *D*, yet another iteration is necessary, this time using a *D* of −0.15 feet. Each iteration provides more cells with “correct” 10-year-return-period *D* values, with the α and *u* Gumbel parameters from the fit that makes the depth “correct” replacing the former parameters. The process continues iteratively, changing the dummy *D* incrementally by −0.05 feet, until all cells have a “correct” estimation of the 10-year-return-period *D*.

The cells having known, positive *D* (i.e., flooded) at only two (i.e., 100- and 500-year) return periods and null *D* values (i.e., flood-free) at the two shortest (i.e., 10- and 50-year) return periods are treated next. These places are less flood-prone than those analyzed previously. For each of these cells taken individually, the Gumbel α and *u* parameters are derived based only on the two return periods and are used to estimate the 50-year return-period *D*. If the calculation results in a positive value, correction is necessary because the cell is known to be flood-free at that return period. In such cases, a dummy 50-year return period *D* of −0.05 feet is assigned for such cells, and the Gumbel distribution is fit once again, this time using this dummy *D*, along with the output for the two *D* values for the same cell. The process continues iteratively, changing the dummy *D* incrementally by −0.05 feet, until all cells have the “correctly” estimated sign of the 50-year-return-period *D*. There is no need to repeat the process for the cells that have 10-year-return-period *D* of the “incorrect” sign, as cells that are not flooded at the 50-year return period will not be flooded at the 10-year return period.

### Parameter estimation for cells not flooded by 100-year return period event

At each cell flooded by the 100-year return period event, the unique α and *u* values are used to extrapolate *D* at that cell for floods of small probabilities (i.e., higher return periods, including 5,000-, 10,000-, 15,000, and 20,000-year), over which the entire study area is assumed to have flooded. The flood elevation of each of these extrapolated extreme periods is calculated as the sum of *D* at that return period and the ground elevation of the corresponding cell. It is necessary to use flood elevation rather than *D* for spatial interpolation because flood elevation is insensitive to differences in surface elevation.

Several spatial interpolation techniques are applied to the study area, separately for each extreme return period (i.e., 5,000-, 10,000-, 15,000, and 20,000-year). A moving average filter is used to impute all missing flood elevation cells in the study area, by experimenting with different window sizes. The dimensions of the final window selected are determined as the smallest that can impute all missing cells, with the same-sized window used for all return periods. Then, because the flood elevation surface of a completely flooded surface should be smooth, a 3 × 3 moving window is run to smooth the flood elevation surface (i.e., reduce undulations over the flooded terrain). Along with the moving average-smoothing, IDW, natural neighbor, and ordinary kriging spatial interpolation techniques are also used (separately) to impute the missing cell values. Assessment of the relative effectiveness of each technique is conducted. The result of the spatial interpolation procedure is a complete set of flood elevations at each extreme return period for each cell in the study area, including those cells for which the values were expunged at the shorter return periods.

After deducting the ground elevation, *D* for the extreme return period events (i.e., 5,000-, 10,000-, 15,000-, and 20,000-year) is used to estimate the flood characteristics in areas unflooded at the 500- and 100-year return periods. Several scenarios are possible. First, for cells that have a positive 500-year *D* (i.e., are flooded) but are unflooded at 100-year (and shorter) return periods, the Gumbel distribution is fit using the 500-year return period *D* along with the spatially interpolated estimates at 5,000-, 10,000-, 15,000-, and 20,000-year return periods, and a dummy 100-year return-period *D* of −0.05 feet. If the resulting estimation of the 100-year return-period *D* is negative, the values are accepted. However, a (falsely) positive 100-year return period *D* calculation requires a refitting using the Gumbel distribution for a 100-year return period *D* of −0.10 feet. Again, if the value is falsely positive, the iteration process continues at incrementally changing dummy values until the 100-year return-period *D* is (correctly) negative (i.e., null, or flood-free).

A second scenario occurs for cells that have a null *D* (i.e., unflooded surface) at the 500-year return period but a positive estimated *D* (i.e., flooded) at the 5,000-year return period. For such cells, the Gumbel distribution is fit using the spatially interpolated estimates at the 5,000-, 10,000-, 15,000-, and 20,000-year return periods along with a dummy *D* of −0.05 feet for the 500-year return period. The iteration process continues analogously to the previous examples, but with a 500-year return-period *D* of −0.10, −0.15 feet, etc. until the 500-year return-period *D* estimate is (correctly) flood-free.

Likewise, the third scenario involves cells with null (i.e., flood-free) *D* at 500- and spatially interpolated 5,000-year return periods. In such cases, the Gumbel distribution is fit using the 5,000-, 10,000-, 15,000-, and 20,000-year return period estimates.

The fourth scenario involves correcting any cells for which the spatially interpolated 5,000-year depth is spuriously less than the Risk MAP-modeled 500-year *D*. In those cases, the Gumbel distribution is fit using the 500-year *D* along with a dummy flood 100-year return period *D* of −0.05 feet. If the resulting 100-year value is (falsely) positive, the fitting process continues iteratively (using −0.10, −0.15 feet, etc.) until the estimated 100-year *D* becomes a negative value.

### Validation of the Gumbel fit and spatial interpolation techniques

Model validation is then performed by statistically comparing the estimated *D* at the 10-, 50-, 100-, and 500-year return periods with the originally available Risk MAP-modeled data. More specifically, the estimated *D* at the 10-, 50-, 100-, and 500-year return periods should be negative in flood-free cells and positive in flooded cells, as represented in the originally available data. Descriptive statistics are presented based on the estimated and original *D* values, where the Gumbel distribution is fit initially with the original available *D* data.

Then, four spatial interpolation methods are implemented (one at a time, separately) to estimate Gumbel parameters (i.e., α and *u*) for cells having zero or only one non-null *D* values (i.e., at the 500-year return period), based on values calculated at cells with two or more non-null values. The validity of the Gumbel estimation of *D* at cells having one non-null value is assessed *via* the descriptive statistics of the difference between the estimated and Risk MAP-modeled value at the known (i.e., 500-year) return period, by spatial interpolation technique.

### Sensitivity analysis

A sensitivity analysis is performed, cell by cell, to check the extent to which the success of the estimation procedure, based on the Gumbel parameters, hinges on the number of “known” *D* values. The model fit is assessed separately *via* descriptive statistics for the complete set of paired predicted vs. known *D* values at a particular return period. At each cell, taken one at a time, if *D* is known from Risk MAP-model-output at 10-, 50-, 100-, and 500-year return periods, the 10-, 50-, and 100-year-return-period *D* values are used to predict the 500-year-return-period *D*. An analogous procedure is used for cells that have known *D* at three return periods. Similarly, the *D* values at 10- and 50-year return periods are used to predict *D* at the 100- and 500-year return periods. In each case, the model fit is assessed separately via descriptive statistics of the paired difference between predicted vs. known *D*.

## Results

### Data cleaning

The data cleaning process described in Section 2.2 is run on the 121,215 cells in the study area. Data cleaning identifies 32 cells with *D* equal to zero (no cells have negative *D*), 3,575 cells for which a shorter return period *D* equals or exceeds a longer return period *D*, and 2,365 cells for which a positive shorter return period *D* is accompanied by a “null” longer return period *D* (Table 2). The original *D* values in these 5,972 cells (4.9% of the initial cells) are thus unused in the analysis because they fail one or more of these data cleaning tests.

**Table 2:** Number of cells in the study area removed by each data cleaning criterion.

**Data cleaning rule**	**Number of cells**
10-year flood depth ≤ 0	13
50-year flood depth ≤ 0	16
100-year flood depth ≤ 0	1
500-year flood depth ≤ 0	2
10-year flood depth ≥ 50-year flood depth	776
10-year flood depth ≥ 100-year flood depth	0
10-year flood depth ≥ 500-year flood depth	2
50-year flood depth ≥ 100-year flood depth	530
50-year flood depth ≥ 500-year flood depth	4
100-year flood depth ≥ 500-year flood depth	2,263
10-year flood depth ≥ 0 and 50-year flood depth is NULL	7
10-year flood depth ≥ 0 and 100-year flood depth is NULL	0
10-year flood depth ≥ 0 and 500-year flood depth is NULL	0
50-year flood depth ≥ 0 and 100-year flood depth is NULL	4
50-year flood depth ≥ 0 and 500-year flood depth is NULL	1
100-year flood depth ≥ 0 and 500-year flood depth is NULL	2,353
**Total**	**5,972**

### Gumbel fitting

Descriptive statistics for the scale (α) and location (*u*) parameters are shown in Table 3. Once the α and *u* parameters are corrected for all cells, they are used to extrapolate *D* for the 5,000-, 10,000-, 15,000-, and 20,000-year return periods in their respective cells.

**Table 3:** Descriptive statistics of α and *u* for the location (cells) flooded by more than one return period in the Metairie, Louisiana, study area.

**Gumbel parameter**	**Mean**	**Standard deviation**	**Minimum**	**Maximum**
α	0.24	0.08	0.08	0.82
*u*	−0.33	0.37	−3.16	0.00

The smallest possible moving-average window that interpolates all flood elevation values at extreme return periods is 31 × 31 cells. Descriptive statistics for the spatially interpolated and smoothed Gumbel parameters are shown in Table 4. A negative value is found for *u* in every cell. The Risk MAP-modeled 500-year *D* spuriously exceeds the spatially interpolated 5,000-year depth in 36 cells (0.03% of the study area), so correction procedures described in Section 2.4 in the “fourth scenario” are implemented.

**Table 4:** Descriptive statistics for α and *u*, after implementing a 31 × 31 moving average and a 3 × 3 moving average, based on extrapolated *D* values of the 5,000-, 10,000-, 15,000-, and 20,000-year return periods, for locations flooded by only one (i.e., 500-year) or no return periods, after removal of spurious cells, for the Metairie, Louisiana, study area.

**Gumbel parameter**	**Mean**	**Standard deviation**	**Minimum**	**Maximum**
α	0.28	0.22	0.07	2.08
*u*	−1.72	1.41	−12.96	−0.39

### Validation

The procedure described in Section 2.5 regarding validation of the distribution is implemented for the case study area. Table 5 shows the descriptive statistics and root-mean-square error (RMSE) of the difference between estimated and Risk MAP-modeled data for cells having at least two non-null *D* values. These results verify that a relatively small amount of error is introduced in the estimation procedure, if it can be assumed that the Risk MAP data are “correct.”

**Table 5:** Descriptive statistics and root-mean-square error for Risk MAP-modeled minus predicted *D*, for cells having two or more originally-modeled *D* from among 10-, 50-, 100-, and 500-year return periods, for Metairie, Louisiana, study area.

	**Mean (ft.)**	**Standard deviation (ft.)**	**Minimum (ft.)**	**Maximum (ft.)**	**RMSE (ft.)**
10-year	0.17	0.21	−0.25	1.58	0.27
50-year	−0.01	0.09	−0.33	0.53	0.09
100-year	0.13	0.07	−0.00	0.85	0.15
500-year	−0.10	0.11	−0.95	0.57	0.14

For cells having only a 500-year Risk MAP-modeled *D*, the relative correspondence between the spatially interpolated estimated 500-year *D* and that from Risk MAP is calculated by spatial interpolation technique. Because of the strong correspondence across spatial interpolation methods, values are expressed in inches (Table 6). Results suggest that the selection of spatial interpolation technique has little impact on the results.

**Table 6:** Descriptive statistics and root-mean-square error for Risk MAP-modeled minus predicted 500-year *D*, for cells having only 500-year return period flood depth, for the Metairie, Louisiana, study area, by moving average (31 × 31) and smoothing (3 × 3), inverse distance weighting, natural neighbor, and ordinary kriging.

**Interpolation technique**	**Mean (in.)**	**Standard deviation (in.)**	**Minimum (in.)**	**Maximum (in.)**	**RMSE (in.)**
Moving average and smoothing	−1.14	1.30	−11.43	6.90	1.73
Inverse distance weighting	−1.12	1.32	−11.43	6.92	1.73
Natural neighbor	−1.11	1.33	−11.43	6.92	1.73
Ordinary kriging	−1.12	1.32	−11.43	6.93	1.73

### Sensitivity analysis

The sensitivity analysis described in Section 2.6 quantifies the rationality of using Gumbel extreme value distribution even as the number of known points decreases to two (Table 7). Results suggest that, not surprisingly, the increased magnitudes of the 500-year *D* leave a wider range from which the estimate can deviate from the actual *D*. Also, it is not surprising that the largest standard deviation of this modeled-vs.-estimated difference occurs for predicting the 500-year *D* when *D* is known at only two return periods. Nevertheless, even in such cases, the RMSE falls within a half-foot.

**Table 7:** Descriptive statistics and root-mean-square error of the difference (Δ) between the Gumbel model-based flood depth (*D*) estimation and Risk MAP-modeled *D*, when using *D* at known return periods to predict *D* at another known return period, for Metairie, Louisiana, study area.

**Scenario**	**Mean (ft.)**	**Standard deviation (ft.)**	**Minimum (ft.)**	**Maximum (ft.)**	**RMSE (ft.)**
Δ 500-year depth using 10-, 50-, and 100-year depth as predictors	0.32	0.22	−0.26	1.87	0.39
Δ 100-year depth using 10- and 50-year depth as predictors	−0.02	0.20	−0.46	1.09	0.20
Δ 500-year depth using 10- and 50-year depth as predictors	0.28	0.38	−0.46	2.65	0.47

## Discussion and limitations

This method offers a means for circumventing the ever-present dilemma of how to ensure high-quality modeling to support planning for preventing, mitigating, and/or adapting to future flood events when little measured data are available, for locations where advanced hydrological and hydraulic modeling has been conducted to determine estimate *D* at multiple return periods. In the case study area in Metairie, Louisiana, only ~5 percent of the cells failed the “data cleaning” tests, which suggests that the modeled data are reasonable. Nearly all of the spurious data occurred when shorter return period *D* exceeds longer return period *D* or longer return period *D* is null.

If it can be assumed that the Risk MAP-modeled data are the “correct” values, the Gumbel distribution-generated flood parameters are shown to be remarkably stable for simulating and imputing *D* for various return periods. The fact that *u* remains negative in all cases verifies that the correction algorithm succeeded in ensuring that all terrestrial cells are not submerged under normal conditions. The much smaller standard deviation for α than for *u* is likely an artifact of the small, homogeneously-elevated study area. As α represents the slope of the Gumbel fit line, each cell in the study area will have a similar relationship between *D* and *P*. This contrasts with *u*, which can have a wider range of values, suggesting that some cells are more susceptible to flooding than others, even within the same neighborhood.

Validation and sensitivity analysis confirm that the method is relatively insensitive to the spatial interpolation technique chosen, at least for this study area. The relatively small errors, as evidenced by the small RMSE values (see Table 5), even for 500-year *D* and even when *D* values for only two return periods are known, are interpreted as evidence that the procedure is successful. The Gumbel distribution is deemed to provide an acceptable result. However, the present work does not consider the uncertainty in the Gumbel parameters. Moreover, the relatively small RMSE values, even between estimated vs. modeled 500-year *D* and even when *D* values for only two return periods are known, imply that *D* can be estimated relatively accurately and precisely. Such estimates can provide engineers and planners with useful information for enhancing infrastructure to accommodate low-frequency, large-magnitude flood events. Although the method is computationally intensive, it can be automated for improved *D* estimates for any location that is “data rich” regarding *D* grids at multiple return periods. Refinements in the modeled data for short or long return periods may allow for further improved understanding of infrastructure needs for accommodating floodwaters.

As with any research, there are limitations to the analysis and interpretation of results. Flood hazard estimation is, by necessity, based on such a limited number of data points, but the availability of model output at only a small number of locations and return periods necessitates use of this technique. Moreover, the rounding of original FEMA-modeled values to the tenth of a foot restricts the precision with which the results can be presented. This method was applied to a relatively limited geographical extent with homogeneous topography. Future work should evaluate the performance of the method across a larger geographical extent with more heterogeneous topography. In addition, the effect of climate change on flood hydroclimatology is not considered (Zhou et al., 2012). Changing climate may alter the log-linear shape of the Gumbel distribution, particularly if forecasts of increasing frequency of extreme precipitation events (Intergovernmental Panel on Climate Change., 2014, p. 8) prove to be accurate. Likewise, differences in local land cover may cause differences in the Gumbel parameters for *D* as a function of return period and in generating a continuous surface using the spatial interpolation techniques. Despite the fact that caution should be exercised in the interpretation of results for these and other reasons, the approach offers an advantageous “next step” in planning for, forecasting, and mitigating the world's most destructive natural hazard.

## Summary and conclusions

Existing *D* grids based on Risk MAP hydrologic and hydraulic model output provide communities with guidance data for anticipating and minimizing flood hazards. However, these depth grids are only available for limited locations and return periods. This study introduces a method for imputing flood depths and elevations for areas considered at low- to moderate-risk, where insufficient flood data are available to characterize the hazard. The method involves fitting the Gumbel extreme value distribution to rasterized flood data of flood depth as a function of annual non-exceedance probability, by cell. The method then uses the Gumbel parameters of scale (α) and location (*u*) to extrapolate flood elevations at extreme return periods for which it can be assumed that the study area is entirely flooded. Spatial interpolation algorithms are used to fill and smooth spatially the areas that are not flooded by the 100-year flood, and Gumbel scale and location parameters are determined for areas with previously uncharacterized or minimally characterized flood hazards. Validation and sensitivity analyses are conducted through comparison with Risk MAP-modeled output. A case study in Metairie, Louisiana, is used to illustrate the technique. For the study area, different spatial interpolation methods produced similar results when compared to Risk MAP-modeled output *D* grids. Validation and sensitivity analyses of the case study illustrate that the method offers improvements in characterization of flood hazard for enhanced flood mitigation planning.

Overall, the method performed well across the study area. The specific findings of the case study include that:

- the presented method is able to characterize flood hazards in areas of low to moderate flood risk; for example, 100-year *D* were predicted for cells with known 100-year *D* with RMSE of 0.15 feet.
- spatial interpolation of extrapolated surfaces functioned well, regardless of technique; for example, 500-year *D* were imputed using spatial interpolation for cells with known 500-year *D* with RMSE of 1.73 inches.
- using 10-, 50-, and 100-year *D* as predictors, the estimated 500-year *D* had an RMSE of 0.39 feet while the estimated 100- and 500-year *D* had an RMSE of 0.20 and 0.47 feet, respectively, when using 10- and 50-year *D* as predictors.

Future availability of longer-return-period *D* grids, such as for the 1,000-year flood, will enhance accuracy of our results. Additionally, because many areas have modeled *D* for only the 100-year return period or for no return periods at all, operationalization of the technique for locations that lack high-quality, modeled *D* at multiple return periods is needed (Shen et al., 2021). Specifically, ratios between the 100-year *D* and the *D* estimated at other return periods, from nearby “data-rich” areas such as Metairie should be calculated as shown here. Then, the ratio between 100-year *D* and *D* at other return periods may be used to derive *D* at other return periods where only the 100-year *D* has been modeled hydrologically (i.e., “data-medium” areas). Then, the relationship between ground elevation and the 100-year *D* can be used to identify the 100-year return period *D* for locations where no hydrological model output is available (i.e., “data poor” areas), based on that from data-rich and data-medium areas. Finally, if such modeling efforts yield plausible results, estimation of *D* for other return periods in “data-poor” areas can be made based on the Risk MAP output from “data-medium” and “data-rich” areas.

## Data availability statement

The datasets presented in this study can be found in online repositories. The names of the repository/repositories and accession number(s) can be found below: https://github.com/adilurrahim/Data-driven_Flood_Hazard_Characterization.

## Author contributions

RM and MR developed the methodology. RM collected and analyzed the data and developed the initial text. MR prepared the code, run the analysis, and edited the text. CF provided original ideas and advice on the overall project methodology and edited the text. RR edited early and late drafts of the text. NB expanded the literature review and edited the text. FO prepared Figure 1 and helped to validate the code. All author contributed to the article and approved the submitted version.
